# How Does a Single Cell Know When the Liver Has Reached Its Correct Size?

**DOI:** 10.1371/journal.pone.0093207

**Published:** 2014-04-01

**Authors:** Nadine Hohmann, Wei Weiwei, Uta Dahmen, Olaf Dirsch, Andreas Deutsch, Anja Voss-Böhme

**Affiliations:** 1 Center for Information Services and High Performance Computing, Technical University Dresden, Dresden, Germany; 2 Department of General, Visceral and Transplantation Surgery, University Hospital Jena, Jena, Germany; 3 Institute of Pathology, University Hospital Jena, Jena, Germany; University of Navarra School of Medicine and Center for Applied Medical Research (CIMA), Spain

## Abstract

The liver is a multi-functional organ that regulates major physiological processes and that possesses a remarkable regeneration capacity. After loss of functional liver mass the liver grows back to its original, individual size through hepatocyte proliferation and apoptosis. How does a single hepatocyte ‘know’ when the organ has grown to its final size? This work considers the initial growth phase of liver regeneration after partial hepatectomy in which the mass is restored. There are strong and valid arguments that the trigger of proliferation after partial hepatectomy is mediated through the portal blood flow. It remains unclear, if either or both the concentration of metabolites in the blood or the shear stress are crucial to hepatocyte proliferation and liver size control. A cell-based mathematical model is developed that helps discriminate the effects of these two potential triggers. Analysis of the mathematical model shows that a metabolic load and a hemodynamical hypothesis imply different feedback mechanisms at the cellular scale. The predictions of the developed mathematical model are compared to experimental data in rats. The assumption that hepatocytes are able to buffer the metabolic load leads to a robustness against short-term fluctuations of the trigger which can not be achieved with a purely hemodynamical trigger.

## Introduction

The liver is a vital organ and its capacity to regenerate and precisely restore its original size is unique among the internal organs of mammals. Partial hepatectomy, especially the resection of two-thirds of the original liver mass is an experimental model for the study of liver regeneration. Partial hepatectomy is well tolerated and the liver grows back to its original size within about 7–10 days in rats and mice [1]. However, how do organs know when they have reached the right size? This question is still under debate [2]. Liver regeneration has been extensively studied, e.g. [3]–[7], but the aspect of size regulation has received little attention. Understanding the crucial factors for liver size regulation has high clinical relevance. Techniques to selectively control liver size could provide new chances for liver transplantation and resection operations [8].

In general, organ size regulation can be driven by organ-intrinsic or by organ-extrinsic factors [9]. The term organ-intrinsic refers to influences related to causes within the concerned organ. Different mechanisms for organ-intrinsic size regulation have been discussed in the literature. Many of the studies choose the Drosophila wing imaginal disc as biological model system [10]–[14]. In these publications, mechanisms based on morphogen gradients, mechanical feedback, or activator-inhibitor systems are discussed that eventually lead to a growth stop. In contrast, organs whose size is primarily determined by organ-extrinsic factors adapt their size to the host by increasing in size when the organ is too small with respect to the organism size or decreasing in size when the organ is too large with respect to the organism size [15], [16]. Since transplanted liver adapts its size to the host [16], a liver size regulation mechanism needs to include an organ-extrinsic factor. As a consequence, the proposed mechanisms for primarily organ-intrinsic size regulation cannot be simply applied to the case of liver regeneration.

There is evidence that the organ-extrinsic trigger of growth after partial hepatectomy is mediated through the portal blood flow. The liver is supplied with blood from the portal vein and the hepatic artery. After two-thirds partial hepatectomy the contribution of portal blood per unit liver tissue increases threefold [6], [17] and the remaining liver grows. In contrast, a deprivation from portal blood by a portal vein ligation leads to a decrease in liver size [18]. Portal blood supply appears to be essential for liver size regulation, but it remains unclear, if either or both the quantity of portal blood [7], [19] and the quality, that is the composition of the blood, are crucial. As regards the quantity of blood, in particular the forces resulting from the portal blood flow, also denoted by the term hemodynamic factors, are discussed as potential triggers of proliferation [20]–[22]. Concerning the quality of blood, the concentration of metabolites in the blood is considered as trigger of proliferation [3], [23], [24]. The resultant work load on the liver is referred to as metabolic load. The hemodynamics as well as the metabolic load hypothesis find support in the literature [3], [20]–[24].

The hemodynamics hypothesis is based on the assumption that an elevation of the shear stress level in the sinusoids, triggers hepatocyte proliferation. The elevated shear stress level is a result of changes in the portal blood pressure. After partial hepatectomy, the portal blood pressure and therefore the pressure difference between portal vein and central vein in the remaining lobules increases [25]. As a consequence, the flow velocity and thus the shear stress in the sinusoids are elevated. Hepatocytes are directly exposed to shear through sieve plates [21]. The shear stress is assumed to trigger hepatocyte proliferation [22], [25]. As liver mass is restored, the shear stress returns to the normal level, such that the trigger for proliferation disappears [21]. Decreased shear stress levels have been proposed to lead to apoptosis [21]. It can be stated that partial hepatectomy has an effect on the hemodynamics, in particular on the shear stress level. However, whether and if so, to which extent proliferation and apoptosis are triggered by shear stress is less clear.

The metabolic load hypothesis is based on the assumption that an increased metabolic load triggers hepatocyte proliferation. The metabolic demands imposed on the liver are mediated through the portal vein flux. The portal contribution of hepatic circulation approximately triples [6], [17] after partial hepatectomy. Hepatocytes sense changes in the metabolic load and react by metabolic adaptations in order to buffer the increased load [6]. Hepatocyte proliferation is assumed to be coupled to the metabolic load on the liver [4]. A principal intracellular mechanism that could contribute to this coupling is described in a mathematical model by Furchtgott et al. [26]. As the liver grows back, the ratio between liver mass and body mass returns to a specific value [3] and the trigger for proliferation vanishes. It can be stated that the metabolic load is altered by a partial hepatectomy but whether and, if so, to which extent proliferation or apoptosis are triggered by an altered metabolic load is unknown.

The metabolic load and the hemodynamics hypotheses are both related to the portal blood flow. It remains unclear, if either or both the concentration of metabolites in the blood and the shear stress are crucial for hepatocyte proliferation and apoptosis after partial hepatectomy. However, the hypotheses differ with respect to their specific feedback mechanisms at the cellular scale. Therefore, a cell-based mathematical model is developed that helps to evaluate the potential effects of these two mechanisms. The chosen model framework is an extension of an interacting particle model which incorporates rules for cell proliferation and death that depend on the metabolic load or on the hemodynamics situation of the cell respectively. By abstracting the principle feedback mechanisms for both hypotheses, a comparison of both hypotheses with the help of mathematical modeling becomes feasible. The modeling approach is validated by close iteration between model and experiments. Thereby our experimental studies are used to decide for reasonable model abstraction and at the same time provide results for the comparison of both hypotheses. It shows that the hepatocytes' capability to buffer the metabolic load leads to a robustness against short-term fluctuations of the trigger which can not be achieved via a purely hemodynamical trigger.

## Material and Methods

On the basis of our experimental studies and data from the literature, reasonable abstractions for a mathematical model are derived. First the performed experimental studies are introduced. Second, results of our experimental studies as well as data from literature are identified that provide the basis for the development of a mathematical model to compare both hypotheses. Then the mathematical model is specified and analyzed. The results of both the experimental studies and the model analysis are given in the Results section.

### 2.1 Animals and experimental methods

#### Ethical statement

All procedures and housing of the animals were carried out according to the German Animal Welfare Legislation. The study was approved by the Thuringian State Office for Consumer Protection (Thüringer Landesamt für Verbraucherschutz).

#### Study design

72 rats were randomly divided into 3 groups: resection of left lateral and median lobe (70%pHx); portal vein ligation of left lateral and median lobe (70% PVL); Sham operation as normal control (Sham-op). Each group contained 4 subgroups (n = 6/subgroup) with different observation times (1 day, 2 days, 3 days and 7 days). Group size was determined according to [27].

#### Experimental animals

Animal experiments were performed in male inbred Lewis Rats (Charles River, Sulzfeld, Germany) within the age of 6–8 weeks and the body weight of 250–300 g.

#### Housing and husbandry

All rats were fed a laboratory diet with water and rat chow *ad libitum* during the experiment and were kept under constant environmental conditions with a 12-h light/dark cycle in a conventional animal facility using environmentally enriched type IV cages in groups of 3–4 rats.

#### Experimental procedures

All surgical interventions were performed at day-time under inhalation anaesthesia. Rats were placed in an anaesthesia induction chamber. Anaesthesia was performed as described earlier [28]. Rats were placed in an anaesthesia induction chamber. Induction of anaesthesia was performed using an isoflurane vaporizer (Sigma Delta, UNO, The Netherlands). Isoflurane (Nicholas Piramal (I) Ltd, London, UK) concentration was 5% and oxygen flow 0.5 L/min. Anaesthesia was maintained with Isoflurane in a concentration of 23% and an oxygen flow of 0.5 L/min to maintain full anaesthesia during the surgical procedure. Postoperative analgesia was achieved by subcutaneous injection of buprenorphine (Temgesic w, Essex Pharma GmbH, Muenchen, Germany) at one dose of 0.01 mg/kg body weight immediately after surgery.

All instruments were cleaned and tip-sterilized after every operation. All rats were subjected to laparotomy via a transverse abdominal incision. A precise vessel-oriented, parenchyma-preserving surgical technique was used in 70%pHx. 70% PVL was performed with the use of 7-0 suture (Prolene, Ethicon) under an operating microscope (Zeiss, magnification 10–25), so that hepatic denervation was avoided and the artery and bile duct were left intact. Sham operation involved laparotomy without ligature. At the end of operation, animals were allowed to recover on a heating pad. To minimize postoperative pain, buprenorphin (0.05 mg/kg body weight) was applied subcutaneously after operation and at an interval of 12 hours over the next day. Postoperatively, animals had free access to water and rat chow *ad libitum*. Animals were monitored daily to record body weight and to assess activity. During the observation time, the clinical condition of rats was observed and judged by using a semi-quantitative scoring system that was described previously [29]. Briefly, rats with normal activity, physiological position, no jaundice, and no signs of bleeding were regarded as healthy (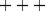); animals showing a weaker activity, hunched back position and/or signs of jaundice or bleeding were regarded as weak (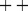); and animals with no spontaneous activity and lying position and signs of jaundice or bleeding were regarded as severely ill (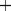). Normalized body weight recovery rate was calculated (body weight after operation/original body weight, expressed as %) and was used as the indicator of recovery after operation. One hour before harvest, *5-bromo-2-deoxyuridine* (*BrdU*, SIGMA-ALDRICH, St. Louis, MO, USA) was injected intravenously in a dose of 50 mg/kg body weight to reveal hepatocellular proliferation. Animals were sacrificed by exsanguination under anesthesia. The remnant liver and every liver lobe were weighed for calculating the recovery of remnant liver mass. Representative histological specimens were collected from all liver lobes.

#### Primary and secondary experimental outcome parameter

Liver weight recovery and hepatocyte proliferation index were taken as primary and outcome parameters. Body weight recovery, clinical chemistry and clinical monitoring were taken as secondary outcome parameters and are not shown here.

#### Remnant liver recovery

Liver weight adaptation (hypertrophy or atrophy) was assessed by calculating the relative ratio between the body weight at the day of operation and the weight of the total liver respectively a given lobe. The following formula was used:

Liver weight adaptation  =  weight of total liver respectively the individual liver lobe/body weight on day of operation * 100%.

The relative fraction of remnant liver mass was calculated using the formula:

Relative fraction of liver weight  =  weight of individual remnant liver weight/calculated total liver weight * 100%.

Mean values and standard variation were calculated for all groups.

#### Hematoxylin-eosin staining (HE staining)

Liver tissue was fixed in 4.5% buffered formalin for 48 h. Sections, 4 um thick, were cut after paraffin embedding. Thereafter, slides were stained with hematoxylin-eosin(HE) for routine histological examination. All slides were digitalized using a slide scanner (Hamamatsu Electronic Press Co., Ltd, Lwata, Japan).

#### Immunohistochemistry (BrdU staining)

Slides were subjected to BrdU-staining for evaluation of hepatocytes proliferation. The staining procedure was based on a modified protocol of Sigma Inc. After deparaffinization and rehydration (Xylene 30 minutes, 100% ethanol 3 minutes, 90% ethanol 3 minutes, 70% ethanol 3 minutes, distilled water 3 minutes, TBS for 5 minutes), tissue sections were treated with prewarmed 0.1% trypsin solution (Sigma, St. Louis, MO) at 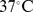 for 40 minutes, followed by denaturation of the DNA with 2 N HCl (Merck, Darmstadt, Germany) at 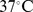 for 30 minutes. In the following step, sections were incubated with 1∶50 monoclonal anti-BrdU antibody (Dako, Hamburg, Germany) at 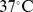 for 1 hour, followed by an alkaline-phosphatase labeled secondary anti-mouse antibody (Power Vision, Immunovision Technologies, USA) for 1 hour at room temperature. Color reaction was performed using the Fast Red Substrate System (sensitive) (Dako, Hamburg, Germany) for 10 minutes. The sections were counterstained with Mayers hemalaun (Merck, Darmstadt, Germany) for 10 seconds, and cover slipped using ImmuMount (Shandon, Pittsburgh, PA). Acquisition of the digital images was performed using the same equipment as the one used during HE staining after BrdU-staining procedures.

#### Assessment of proliferating index

All BrdU-staining slides, containing liver tissue from 4 different lobes, were entirely and quantitatively analyzed using software HistoCAD VirtualLiver (Fraunhofer Mevis, Bremen, Germany). The results are showed as percentage.

#### Statistical methods

The data, expressed as mean
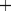
standard deviation, were analyzed using Sigmaplot 10.0 (Statcon, Witzenhausen, Germany). Differences between paired groups were analyzed using the two tailed paired samples from the Students t-test and multiple groups were compared using the one way independent analysis of variance (ANOVA) test. Differences were considered significant if p-values of less than 0.05 were obtained.

### 2.2 Basic abstractions for mathematical modeling

In order to build a mathematical model for size regulation of the regenerating liver after partial hepatectomy, some basic specifications and abstractions of the biological scenario are required.

#### Liver mass recovers initially via compensatory growth of liver lobules

**Figure 1:**
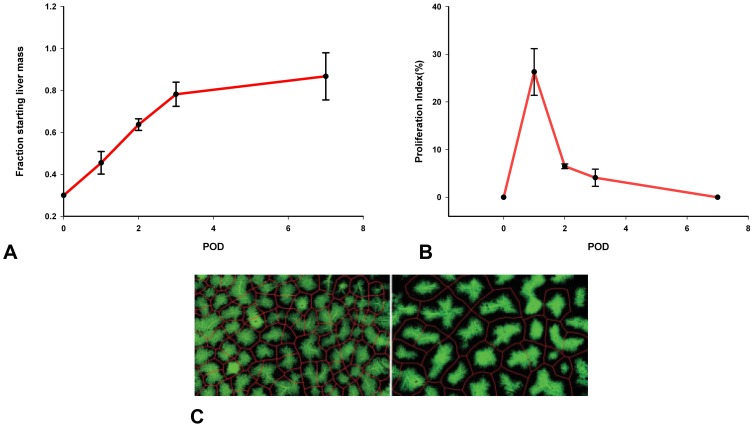
Experimental data for liver regeneration after partial hepatectomy in rats. **A** Total liver mass grows back to its original individual size after partial hepatectomy. Depicted is the time course of liver mass regeneration after partial hepatectomy as the relative fraction of remnant liver mass (fraction starting liver mass) at different postoperative days (POD). **B** Kinetic of proliferation demonstrates a peak on first postoperative day (POD). Depicted is the proliferating index (PI) for the remaining liver lobules after 70% partial hepatectomy. **C** Liver lobules enlarge after partial hepatectomy [30]. Depicted is the retrograde filling of the hepatic sinusoids through the central veins with green fluorescent resin. The lobular borders are shown in red. (left) Liver lobular structure in control rat. (right) Liver lobular structure at four days after partial hepatectomy in rats (identical scale left and right).

The derived basic abstractions are made on the basis of our experimental studies and data from the literature. Figure 1 summarizes the results of our study on 70% *partial hepatectomy (pHx)* (A and B) as well as results of a study by Papp et al. [30] (C). The latter study indicates that liver growth is driven by lobule growth rather than by change of lobule number as illustrated in Figure 1C. The initial enlargement of liver lobules by comparing the liver lobular structure in control rats and at four days after partial hepatectomy is shown in Figure 1C [30]. At a later remodeling phase liver lobes reorganize into lobules of normal size via restructuring [5]. This phase is not considered in the present study. The sections of such lobules are idealized as hexagons or even circles. The hepatocytes are arranged along sinusoids spanning the distance from the portal to the central vein such that each hepatocyte occupies a unique position along a one-dimensional axis [31]. As spatial differences in the blood flow pattern are expected only along this axis between the blood inflow area at the portal vein and the blood outflow area at the central vein, a one-dimensional mathematical model is sufficient to describe the growth of a liver lobule. Figure S1 provides an illustration of this abstractions towards a one-dimensional model.

#### Liver size is modulated by hepatocyte proliferation and apoptosis

The initial growth phase of liver regeneration is dominated by hepatocyte proliferation. In the performed experimental study, the hepatocyte proliferation index is recorded in order to specify the contribution of hepatocyte proliferation to liver size regulation. The observed proliferating index for the remaining liver lobes after 70% partial hepatectomy is depicted in Figure 1B. According to [30], cell volume changes do not reach a significant level and can thus be neglected. Hepatocytes constitute 80%–90% of liver mass and are the first cells to proliferate after partial hepatectomy [6]. Thus, hepatocytes have an outstanding role during the early phase of regeneration and the model can be restricted to that one cell type. As changes in the size and thus in the mass of an individual hepatocyte can be neglected, liver size is equivalent to liver mass. In conclusion, the liver size is proportional to the lobule size which can be approximated by means of the number of hepatocytes in a one-dimensional lobule representation.

#### Hepatocyte proliferation and apoptosis are triggered by blood borne factors

**Figure 2:**
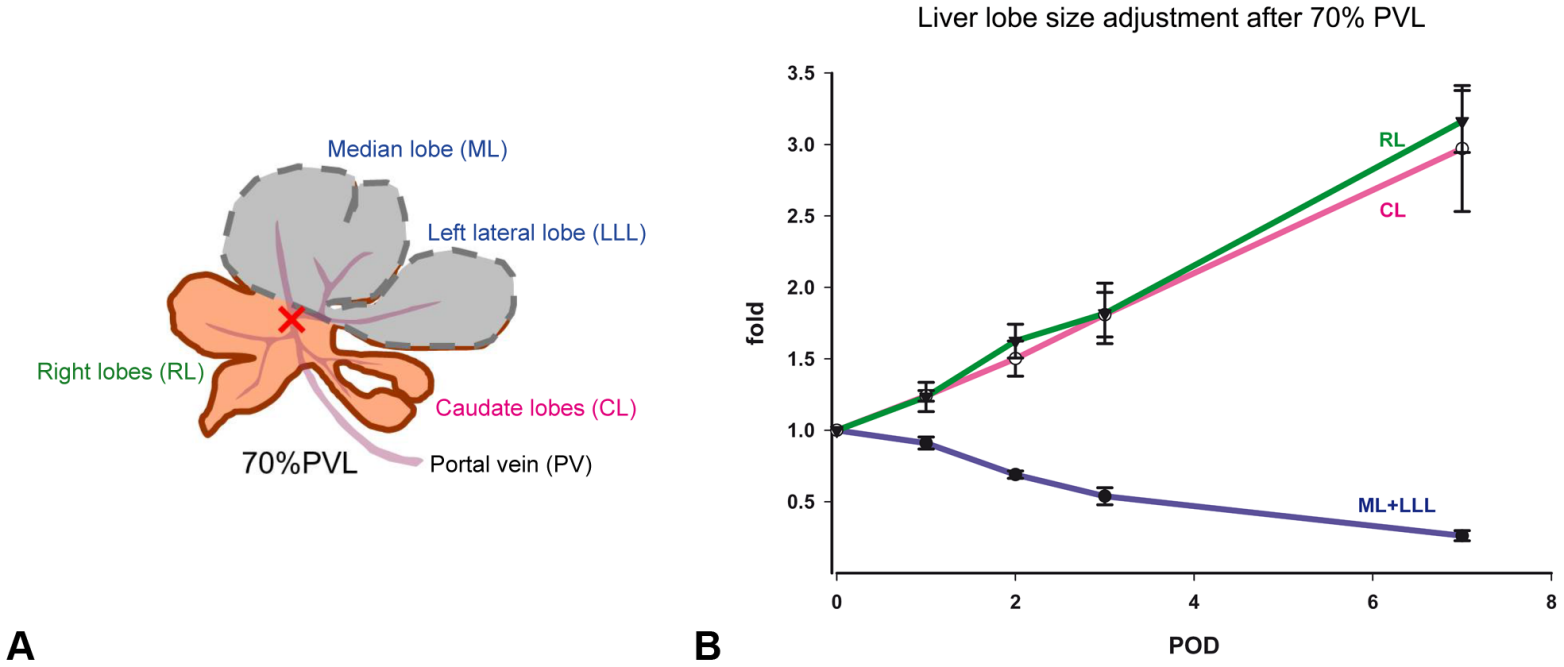
Liver lobe growth is regulated by portal blood supply. Portal vein ligation results in hyperperfusion of right lobes (RL)and caudate lobes (CL) and hypoperfusion of median and left lateral lobes (ML+LLL). The observed growth in the respective lobes correlates with the corresponding perfusion changes. (A) Rat liver anatomy and illustration of the surgical procedure for the portal vein ligation (PVL). Depicted is the positioning of the PVL (red cross). Ligated lobes are marked in gray. In case of a 70% pHx the lobes marked in gray are resected. (B) Liver lobe size adjustment at different postoperative days (POD) after of 70% PVL. Ligation of left lateral and median portal vein (70% of liver mass) results in lack of portal perfusion in the ligated 70% of the liver and hyperperfusion of right and caudate liver lobe, similar to the hyperperfusion observed after 70% pHx. Liver lobes adjust their weight according to the portal supply: Ligated lobes shrink to one third of their original weight whereas non-ligated lobes experience a 3-fold increase of their original weight, as seen after 70% pHx. Depicted is the time course of liver mass adaption after portal vein ligation as the relative liver lobe mass in proportion to the initial lobe weight (fold) at different postoperative days (POD).

Organ-extrinsic factors play a major role in liver size regulation. This becomes apparent by a positive correlation between organ and organism mass [32]. In order to test whether portal blood supply is crucial for liver lobe size regulation, *portal vein ligation (PVL)* experiments are performed. In Figure 2A, the principal surgical procedure of the portal vein ligation is illustrated. In Figure 2B, our experimental data found for liver lobe size adjustment after portal vein ligation at different postoperative days is depicted. It shows that portal blood flow is crucial for the corresponding growth response of a particular liver lobe. As a consequence a representation of the blood flow is included into the mathematical model. Blood flow in a liver lobule is directed from the portal vein to the central vein. It has to be considered that differences in hemodynamics and in the concentration of metabolites occur between the blood inflow area near the portal vein and outflow area near the central vein and as such a spatial model is required.

#### Shear stress and the concentration of metabolites are both potential triggers for hepatocyte proliferation and apoptosis

**Figure 3:**
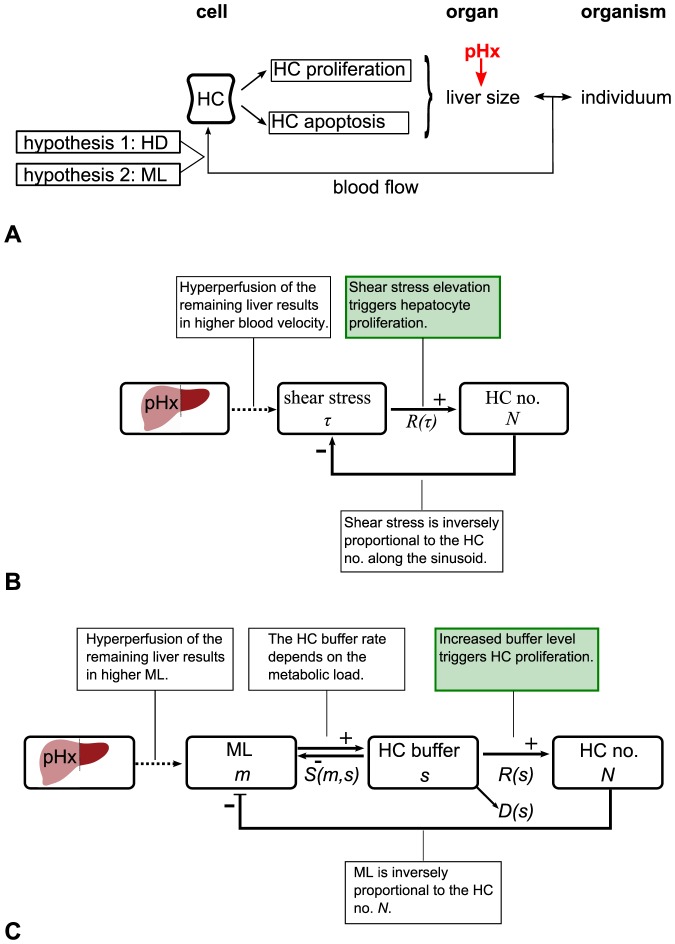
Feedback mechanisms of liver size regulation after partial hepatectomy. (A) Sketch of hypothesized mechanisms of liver size regulation after partial hepatectomy. Liver size regulation is modulated by hepatocyte (HC) proliferation and apoptosis. A partial hepatectomy (pHx) alters liver size and therefore the relation between the organ and the organism. As a consequence, changes in the portal blood flow are observed. HCs respond to portal blood flow changes. The two hypothesized triggers for proliferation or apoptosis that relate to the portal blood flow are the altered metabolic load (ML) per HC and the altered hemodynamics (HD). (B) Feedback mechanism for the HD hypothesis. The main assumption is marked in green. The partial hepatectomy results in a change of the shear stress level which triggers proliferation and therefore affects the hepatocyte (HC) number 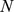. (C) Feedback mechanism for the ML hypothesis. The main assumption is marked in green. The partial hepatectomy alters the metabolic load 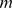 per hepatocyte (HC) and hence the intracellular buffer level 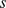. The functional relation between the buffer rate 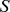 and 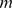 is illustrated in Figure S3B. The intracellular buffer 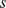 affects the HC number 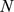. The according growth rate per hepatocyte is given by 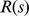, see Figure S3D. At the same time, HCs degrade metabolites from the intracellular buffer at the rate 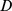 which depends on 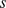, see Figure S3C.

Liver size regulation is modulated by hepatocyte proliferation and apoptosis, that are triggered by blood-borne factors. The two hypothesized triggers that relate to the portal blood flow are the altered *hemodynamics (HD)* and the altered *metabolic load (ML)* per hepatocyte. Figure 3A illustrates the hypothesized mechanisms of liver size regulation after partial hepatectomy. The HD hypothesis is based on the assumption that an elevation of the shear stress level triggers hepatocyte proliferation. The feedback mechanism for the HD hypothesis is depicted in Figure 3B. The wall shear stress, a measure of force per unit area is inversely proportional to the sinusoid length and thus to the hepatocyte number along the sinusoid, see (3) in the section Mathematical model.

The ML hypothesis is based on the assumption that an increased metabolic load triggers hepatocyte proliferation. The feedback mechanism for the ML hypothesis is depicted in Figure 3C. Hepatocytes synthesize, degrade, and store metabolites [33]. In the following, the uptake and internal processing of metabolites by hepatocytes is referred to as buffering, regardless if metabolites are actually stored or synthesized over a period of time. Each process that leads to a reduction of intracellular buffered metabolites is subsumed as degradation. After partial hepatectomy hepatocytes perform all essential functions needed for homeostasis, including glucose regulation, synthesis of many blood proteins, secretion of bile and biodegradation of toxic compounds. Little disturbance is observed in the metabolic functions after partial hepatectomy, [5]. This means, firstly, that hepatocytes sense changes in the metabolic load by the remaining hepatocytes and, secondly, that hepatocytes adapt to these changes. Therefore, it is assumed that the buffer rate, as well as the degradation rate adjust to a certain extent. If the intracellular buffer level still exceeds a specific value, proliferation is assumed to be triggered. The hepatocyte capability to buffer as regarded here has two effects: the temporal balancing of upcoming workload and the interception of workload peaks.

#### The hypothesized mechanisms are distinguished on the basis of experimental data on the organ scale

The animal experiments consist of monitoring total liver weight and liver lobe weight recovery after 70% partial hepatectomy and 70% portal vein ligation. After partial hepatectomy, the remaining liver lobe mass triples, see Figure 1A in order to restore the original size (see also [3], [6]). As a consequence, a tripling of original lobule size and the hepatocyte number is assumed. In several studies an intermediate over-shooting of the regenerative response is reported [7], [34]. As this observation is not generally accepted, it is not used as a criterion in this work, but will be discussed on the basis of the simulation results. The time span in which the liver restores its original mass up to 90% is about 7 days in rats, see Figure 1A. An increase of hepatocyte apoptosis is observed in a later phase of regeneration after partial hepatectomy [35]. It is tested whether an according slight decrease in the cell number can be observed in the mathematical model.

The model should be robust against *short-term perturbations (STP)* in the trigger. For instance the intake of food has a transient effect on both, the liver metabolic load and hemodynamics [36], but has no effect on liver size. Therefore, it is tested whether the hepatocyte number changes in the mathematical model in case of short-term perturbations.

In order to test the applicability to other experimental observations the case of a decreased portal blood flow is taken as an example. A portal vein ligation leads to decreased portal blood flow and results in an according liver size adaption. Figure 2 shows the results of the 70% portal vein ligation study performed here. The observed growth response in different liver lobes correlates with the corresponding perfusion changes. This observation agrees with the findings in [18]. With regards to the mathematical model, it is tested whether a decrease in cell number can be observed that scales with the extent of portal flow decrease.

### 2.3 Mathematical model

The mathematical model implements the hypothesized liver size regulation mechanisms after partial hepatectomy, see Figure 3. The model represents a row of equally sized hepatocytes along a sinusoid between portal and central vein. The chosen model is based on the model class *interacting particle system (IPS)* [37]. IPS are continuous-time models and describe local interactions of spatially distributed individuals on a discretized space. They have already been successfully applied to interacting cell systems [38].

**Figure 4:**
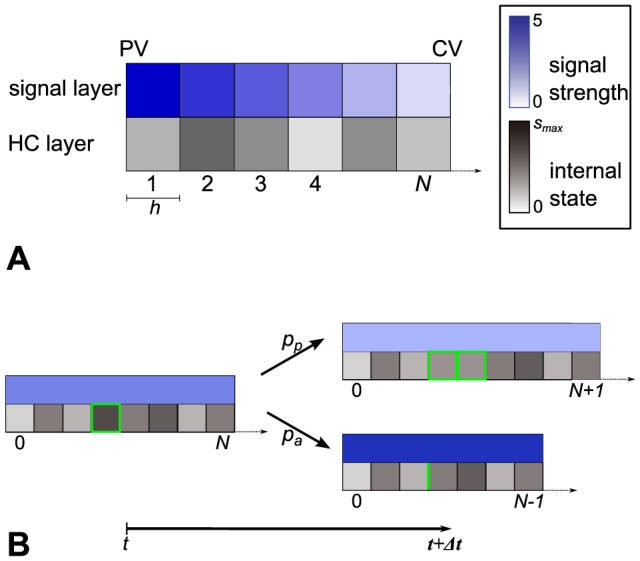
State of the hepatocyte and the signal layer and temporal dynamics in the mathematical model. (A) The one-dimensional hepatocyte (HC) layer represents a row of 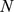 hepatocytes along a sinusoid between *portal vein (PV)* and *central vein (CV)*. The space of the hepatocyte layer is discretized by the constant hepatocyte diameter 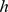. Each hepatocyte possesses an internal state 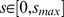. The signal layer is considered to be a one-dimensional continuous domain, which is discretized for simulation purposes. The signal strength at a given position is expressed by a real number. The internal state and the signal strength are illustrated here by a color code. (B) Each cell can either proliferate, survive or undergo apoptosis. The model is computationally analyzed with the help of simulations. Each simulation step, a cell is chosen randomly and proliferation, survival and apoptosis are assigned a probability which depends on the signal strength and the internal state. A proliferation event (upper right) is performed with probability 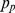. A new cell is inserted right to the mother cell. The mother and daughter cells are marked in green. An apoptosis event (lower right) is performed with probability 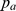. Gaps resulting from apoptosis events are closed by rearrangement. The previous position of the cell is marked in green. The state of the signal layer is updated. Here, the update is illustrated using the HD model as an example. In this model the overall signal strength is inversely proportional to the cell number 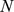, see (3).

Here, the focus is on the influence of a blood-borne signal on the cellular behavior. Therefore the model is adapted to consist of two one-dimensional layers, the hepatocyte and the signal layer. In Figure 4A the state of these layers in the mathematical model is depicted. The hepatocyte layer represents the hepatocytes aligned along the sinusoid. This layer is discretized by the hepatocyte diameter 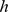. The total number of hepatocytes along the sinusoid in the model is denoted by 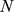. Each cell possesses a position 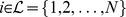. The overall length of the hepatocyte layer is 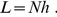
(1)


This corresponds to the radius of a lobule. The cell number 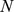 is variable under the dynamics. The classical IPS framework is extended by an optional cell internal state 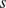. The internal state is utilized as a buffer for metabolic load as described later. There is no internal state in the HD model.

The signal layer represents the strength of the blood-borne signal and constitutes a further extension of the classical IPS framework. It is continuous, but discretized according to the hepatocyte layer for simulation purposes. The length of the signal layer adapts to the hepatocyte layer and is hence 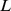. The state of the signal layer at a given position 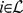 represents the signal strength, that means either the concentration of metabolites 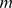 or the shear stress level 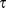 and is expressed by a positive real number. The state of the signal layer is referred to as external state. The state of the mathematical model is completely described by the vector 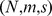 or 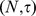, respectively.

The temporal dynamics in the mathematical model are depicted in Figure 4B. Each cell can either proliferate, survive or undergo apoptosis. The growth rate per hepatocyte is denoted by 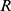. A positive growth rate per hepatocyte can be understood as proliferation rate. A negative growth rate per hepatocyte can be understood as apoptosis rate. The functional dependence of 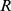 on the external and the internal state is specific for the metabolic load and the hemodynamics hypothesis and is described below, see equations (4) and (9). A proliferation or apoptosis event is associated with a rearrangement of cells. In case of a proliferation event a new cell is inserted to the right of the mother cell. Cells located on the right side of the new cell are shifted by one position. In the case of apoptosis, gaps resulting from apoptosis events are closed by rearrangement, see Figure 4B. This procedure is phenomenologically well motivated and corresponds to mechanisms described in the literature, e.g. [39].

The model is computationally analyzed with the help of simulations. The simulation algorithm is an adaption of the classical Gillespie algorithm to IPS, as developed in [40]. In each simulation step, a cell is chosen randomly. For the chosen cell, each event, proliferation, survival and apoptosis, is assigned a probability which depends on the corresponding rate 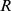. The probabilities (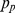), (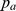) and (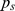) per time step of duration 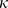 are determined by 
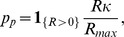


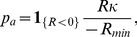
(2)

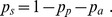



Here, 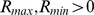 are the maximum proliferation and apoptosis rates, respectively. In order to ensure that 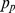 and 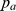 are well-defined such that 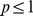, 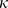 has to be chosen sufficiently small. The specific choice of 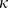 and thus the time discretization in the simulation is explained in the Analysis. In the next section, the mathematical model is made specific for the two hypotheses.

#### Hemodynamics model

The implementation of the hemodynamics (HD) hypothesis in the presented mathematical model is denoted by the expression HD model. All hepatocytes in the hepatocyte layer directly sense the shear stress and respond by proliferation or apoptosis. Hepatocytes in the hepatocyte layer are assumed to have no internal state in the HD model.

In the HD model, the signal that constitutes the signal layer is the wall shear stress. The feedback mechanism in the HD model is depicted in Figure 3B. In order to determine the shear stress at the position of a particular cell along the sinusoid, the Hagen-Poiseuille equation is utilized, assuming that the sinusoid is a cylindrical tube with inelastic walls and blood is a Newtonian fluid whose flow is steady and laminar. According to this equation the wall shear stress 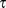 is homogeneous along the sinusoid. The Hagen-Poiseuille equation states 
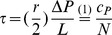
(3)


where 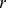 is the sinusoid radius, 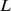 is the sinusoid length, 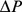 is the pressure difference over the sinusoid and 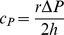. The sinusoid radius 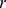 and the hepatocyte diameter 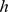 are assumed to be constant. The increase of the shear stress level after partial hepatectomy is the result of an increase in the pressure difference 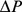. From equations (1) and (3), it follows that there is an inverse proportionality between the total number of hepatocytes and the wall shear stress at one position at the sinusoid.

The function describing the relationship between the shear stress as a signal and the resulting growth rate per hepatocyte is denoted by 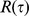 (see Figure S2). The choice of 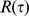 is driven by the following considerations. The shear stress value under normal conditions is denoted by 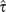. Values higher than the normal value 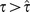 lead to a positive growth rate per hepatocyte 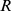, whereas, values lower than the normal value 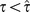 lead to a negative growth rate per hepatocyte 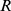. The relationship between the growth rate per hepatocyte and the shear stress level is assumed to be linear. The slope 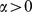 controls the sensitivity to deviations from the normal value 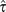. The growth rate per hepatocyte is limited by 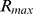 and 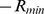. The most elementary function with the desired properties is 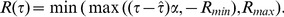
(4)


#### Metabolic load model

The implementation of the metabolic load (ML) hypothesis in the mathematical model is referred to as the ML model. Hepatocytes respond to an increased metabolic load by proliferation and to a decreased load by apoptosis. The feedback mechanism for the ML model is depicted in Figure 3C. The key difference to the the feedback mechanism for the HD model is the intracellular buffer capacity. At each time point the ML model is in a state 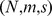. The external state 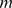 represents the concentration of metabolites. In order to model the hepatocyte capacity of buffering metabolic load an intracellular buffer level for metabolites 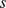 is assigned to each hepatocytes as an internal state. The intracellular buffer level is limited by 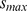.

The metabolites that enter at the PV are transported through the sinusoid by the blood with a constant velocity. Under normal conditions it is assumed that the incoming amount of metabolic load equals 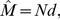
(5)


where 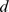 is the amount hepatocytes buffer under normal metabolic load conditions. As a consequence, under normal metabolic load conditions the metabolic load is fully buffered by the present hepatocytes. The concentration profile of metabolites along the sinusoid at a certain time point depends on the relation between transport velocity and the buffer rate of metabolites by hepatocytes. In this study, two special cases are contrasted. First, it is assumed that the transport rate is small compared to the uptake rate, such that a concentration gradient evolves. Second, it is assumed that the transport rate is large in comparison with the uptake and degradation rate, such that the spatial distribution of metabolites in the blood can be considered uniform.

In the first case, the signal layer is discretized. Metabolites are transported stepwise through the signal layer, which has periodic boundaries. Thus, at the source it is 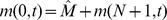. The buffer rate 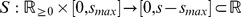 at the position of a particular hepatocyte is given by 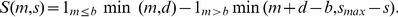
(6)


The value of 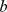 in the definition of 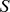 has to be chosen such that under a normal metabolic load condition all cells, even the cell at the source, buffer an amount equivalent to 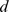. This can be achieved by choosing 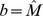.

In the second case the metabolic load is distributed uniformly and thus no peak at a particular position is seen. Therefore, choosing 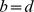 already ensures that under normal conditions all cells buffer an amount equivalent to 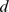. The buffer function simplifies to 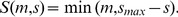
(7)


The degradation rate 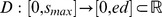 depends in both cases on the intracellular buffer as follows 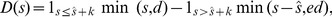
(8)


with 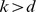 denoting a tolerance range for changes form the normal buffer level 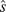. If the intracellular storage exceeds the level 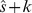 the degradation rate adjusts and can be increased up to a factor 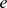, denoting the potential additional degradation 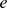.

The coupling of cellular behavior on the signal strength, is given by the growth rate per hepatocyte 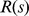


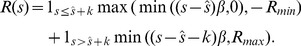
(9)


It is assumed that an increase up to 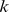 above a normalized value 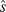 does not lead to positive growth. The maximal growth rate per hepatocyte 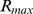 and minimal growth rate per hepatocyte 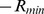 as well as 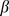, the sensitivity to deviations from the normal value 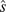 are included as parameters. The described specifications for the ML model are illustrated in Figure S3.

### 2.4 Analysis

In order to analyze the mathematical model, computer simulations are performed. The cell number in time and the proliferation events in space and time are evaluated for the metabolic load (ML) and hemodynamics (HD) models in different scenarios. In this section the procedure to analyze the model is described. The results of this analysis follow in the Results section.

#### Parameter specification

The temporal scale of the model is determined by the the maximal 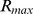 and minimal growth rate per hepatocyte 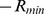. In the HD model, as well as in the ML model, it is assumed that 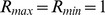. The temporal scale is thus defined in days, such that one time unit in the model will be interpreted as one day. First, the spatial and temporal discretization of the model is specified. The hepatocyte layer is discretized by the hepatocyte diameter 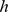, which is normalized to 1. In order to specify the time discretization in the simulation, the time step duration 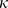 is considered, see (2). The procedure for determining 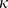 taking the HD model as an example is as follows. A parameter sweep over 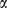, the sensitivity to the deviations from the normal value 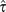, is performed for decreasing values of 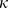. As soon as the result only depends on the parameter 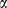 and not on 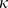, it can be assumed that artefacts due to the time discretization can be neglected. As a result, a value of 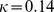 is chosen here. It proves that this value produces no artefacts in the ML model as well.

The parameters in the HD model that need to be specified are the shear stress value under normal conditions 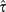 and the sensitivity to deviations from this normal value 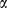, see (4). The value 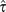 is normalized to 1 such that 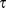 gives the relative change compared to the normal shear stress level in the liver. The parameter 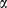 is left as a free parameter.

The parameters in the ML model that need to be specified are 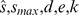 and 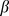, see (6)–(9). The amount which hepatocytes buffer under normal metabolic load conditions is denoted by 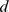 and is normalized to 1. The normal buffer level 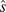 and the buffer capacity 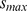 are assumed to be 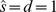 and 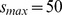, such that the normal buffer level equals the amount hepatocytes buffer under normal metabolic load conditions and and effects of a limited buffer capacity do not occur. The potential additional degradation 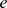, the tolerance range for changes form the normal buffer level 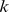 and 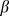, the sensitivity to deviations from the normal value 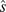, are left as free parameters. All specifications of the model are summarized in Table 1.

**Table 1:** Model parameters for the simulations of the HD and the ML model.

	HD	ML (non-uniform)	ML (uniform)
spatial discretization	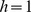		
temporal discretization	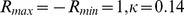		
constant parameters	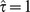	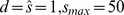	
free parameters	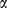	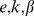	
all scenarios	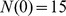		
	-	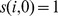	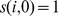
(pHx)	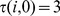	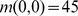	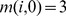
(PVL)	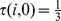	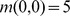	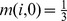
(STP)	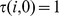	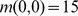	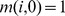
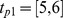	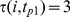	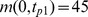	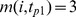
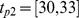	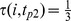	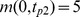	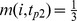

For a selected parameter set, the simulation results in the scenarios increased portal blood flow (pHx), reduced portal blood flow (PVL) and short-term perturbations by fluctuations in the portal blood flow (STP) are studied. For each scenario the state of the cell and the signal layer at initialization is given.

#### Simulation procedure and chosen scenarios

Three scenarios are considered here. First, the scenario partial hepatectomy (pHx) is analyzed. As an initial step, a general sensitivity analysis with respect to the free parameters is performed for the HD as well as for the ML model. As a second scenario, the robustness against short-term perturbations (STP) is examined by applying fluctuations in the portal blood flow. The applicability of the model to the case of decreased portal blood flow is tested by the second scenario denoted as (PVL). The simulation algorithm comprises an initialization and the actual simulation. The model is initialized at 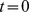 representing the situation at initiation of the different scenarios. With regards to the hepatocyte layer, this means in all scenarios a cell number of 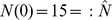 is chosen, which corresponds approximately to the hepatocyte number along the sinusoid in an average liver lobule. In the scenario (pHx), the portal blood flow and thus the shear stress level in the signal layer triples. In case of the HD model, this means 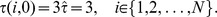



With regards to the ML model with the non-uniform signal layer, it is 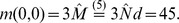



In the ML model with the uniform signal layer, the same quantity of metabolic load is uniformly distributed over the existing cells 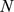 such that 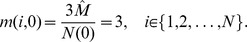



The internal state of each hepatocyte at the initialization is 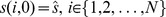 in both alternatives of the ML model. The initial state of the model for (pHx), as well as for the scenarios (STP) and (PVL) is described in Table 1.

Each simulation is performed for 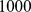 simulations steps or 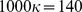 time units, respectively. As the time units are interpreted as days, this number is considered to be sufficient as observation time. Each simulation step consists of 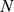 selection steps, whereby the following tasks are executed.

- for each selection step:
  (i) randomly choose one of the 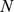 cells
  (ii) only for ML model: buffering (see (6) or (7) ) and degradation (see (8) )
  (iii) determination of proliferation and apoptosis rates (see (4) or (9) )
  (iv) performance of proliferation or apoptosis event depending on (iii)
- for each simulation step: update the signal layer

For each scenario, 100 simulations are performed.

#### Evaluation criteria

The cell number per time averaged over all simulations, the spatial distribution and the frequency of proliferation and apoptosis events are recorded and compared to the experimental data given in the Material and methods section. Specifically, a simulation result is accepted as being in accordance with the experimental results if the following applies

(O1) tripling of *hepatocyte (HC)* number in case of (pHx),
(O2) the time until 90% of the final HC number is achieved can be scaled to about 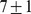 time units, the according time point is denoted by 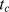

(O3) decrease in HC number at a later simulation time
(O4) no change in HC number in case of (STP), and
(O5) decrease in HC number in case of (PVL).

The parameter set for which the scenarios (STP) and (PVL) are performed is chosen on the basis of (O1)–(O3). The final HC number is said to be achieved, if the HC number has been stable for at least 10 time units.

The choice of a spatial model has been motivated before in this section. Nonetheless, the derivation of the HD and the ML model has revealed that in the HD model as well as in one special case in the ML model the signal layer is uniform. In these cases, an ordinary differential equations system can be set up, which provides additional theoretical results, such as the expected hepatocyte number in the steady state as well as a stability analysis of the steady state. Notice that, the analysis of the temporal dynamics of the HD and the ML model is performed by means of simulations of the cell-based model, since this analysis can only be accessed by numerical methods.

#### Ordinary differential equations approximating the hemodynamics model

As the shear stress is modeled spatially homogeneous, the temporal rate of change of the expected hepatocyte number can thus be approximated by the following ordinary differential equation. Due to spatial uniformity it is 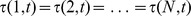 such that 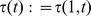 can be defined. 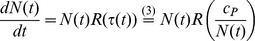



Its steady states and their stability can be analyzed. It shows that the only non-vanishing steady state is 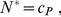
(10)


which is stable, see Text S1 for details. Note that 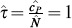. Assuming, 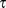 is elevated by a factor 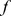 such that 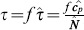, then the steady state is 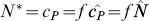.

The expected HC number in the steady state is in accordance with the validation data (O1) and (O2).

#### Ordinary differential equations approximating the metabolic load model

In case of the ML model with a uniform signal layer the following ordinary differential equations system can be derived. Due to spatial uniformity it is 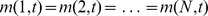. It is additionally assumed that 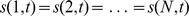. Therefore, it can be defined 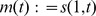 and 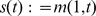. 
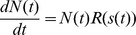


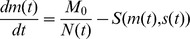


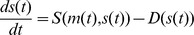


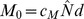



The only non-vanishing steady state of this system is 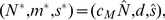
(11)


which is stable, see Text S1 for details. Note that 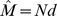. That means, 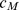 gives the factor by which the metabolic load is elevated above the normal value. The expected HC number in the steady state is in accordance with the validation data (O1) and (O2).

## Results

### 3.1 Experimental results

All animals tolerated the surgical procedure well and were included in data analysis. No adverse events were encountered. Maximum body weight loss was observed on postoperative day 3 and reached 10% after 70% pHx and 5% after PVL and sham operation. Body weight recovery in all 3 groups of animals started on postoperative day 4. Animals subjected to PVL and sham reached their starting weight with 5 days, whereas animals subjected to pHx only reached 90% of their original body weight on postoperative day 7. The data points are depicted in Figures 1A and 2. Figure 1B shows that the hepatocyte proliferation index after 70% pHx peaked on postoperative day 1 (23
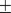
8%) and declined thereafter. The raw data can be found in the Dataset S1.

### 3.2 Simulation results

The simulations for the HD and the ML model are performed according to the procedure described in the Analysis.

#### Hemodynamic model simulations

**Figure 5:**
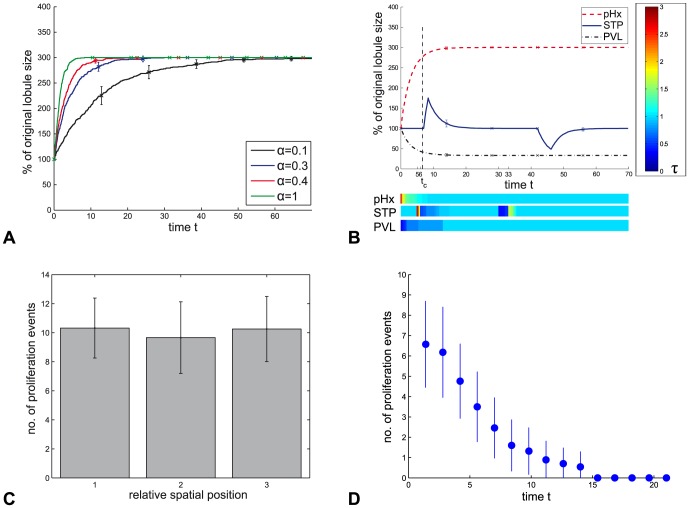
Simulation results of the HD model. **A** Temporary progression of the lobule size in dependence of 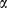, which is the sensitivity of the proliferation response to deviations from the normal shear stress value 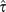. **B** Temporary progression of the lobule size in the simulation with 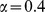 (red curve in **A**) in the scenarios (pHx), (STP) and (PVL) as given in Table 1. The shear stress strength is illustrated by a color-code for the different scenarios. The time point at which compensatory growth is 90% completed is marked with 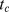. **C** Spatial distribution of proliferation events for the scenario (pHx). For each time point, the hepatocyte layer is equally divided into three sections displayed on the horizontal axis. The proliferation events are recorded for each section over the entire simulation time and are depicted as number of proliferation events for different relative spatial positions. **D** The frequency of proliferation events for scenario (pHx) is depicted as number of proliferation events per time point.

In Figure 5 the simulation results of the hemodynamics (HD) model are summarized. First, results of the sensitivity analysis in the scenario (pHx) are reported. It is observed that 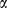, the sensitivity to deviations from the normal value 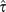, has no effect on the final number of hepatocytes but on the temporal progression of the HC number increase. In Figure 5A, the result for 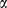 varied within the rage 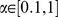 is depicted. When choosing 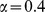, compensatory growth is completed to an extent of 90% within the time interval 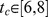. The sensitivity analysis shows that the final HC number is robust against variations of 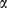 and that 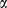 can be chosen such that the temporal progression of the growth process is in accordance with experimental observations.

As a next step, the results for all scenarios (pHx), (PVL) and (STP) are described. They are implemented with 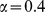 and as stated in Table 1.

In the simulation of (pHx), the HC number triples. The increase in HC number is rapid and the number of HCs is stable as soon as the final number of HCs is achieved, see Figure 5B. The shear stress level in the signal layer normalizes correspondingly, see Figure 5B horizontal bar below. Compensatory growth is completed to an extent of 90% on average within 6.6 time units. The proliferation events are uniformly distributed along the sinusoid, see Figure 5C. The frequency of proliferation events is depicted in Figure 5D. The size is regulated very precisely, that means there is no overshooting. There are no apoptosis events observed.

The scenario (STP) shows that the model is not robust against short-term perturbations. A (STP) leads to an instantaneous change in the HC number. The HC number normalizes to the initial number as the shear stress level normalizes, see Figure 5B.

The HC number decreases in the simulation of (PVL). The decrease is scaled to the degree of shear stress level decline. During the simulation of (PVL) the HC number reaches a stable state and the shear stress level normalizes correspondingly, see Figure 5B.

#### Metabolic load model simulations

**Figure 6:**
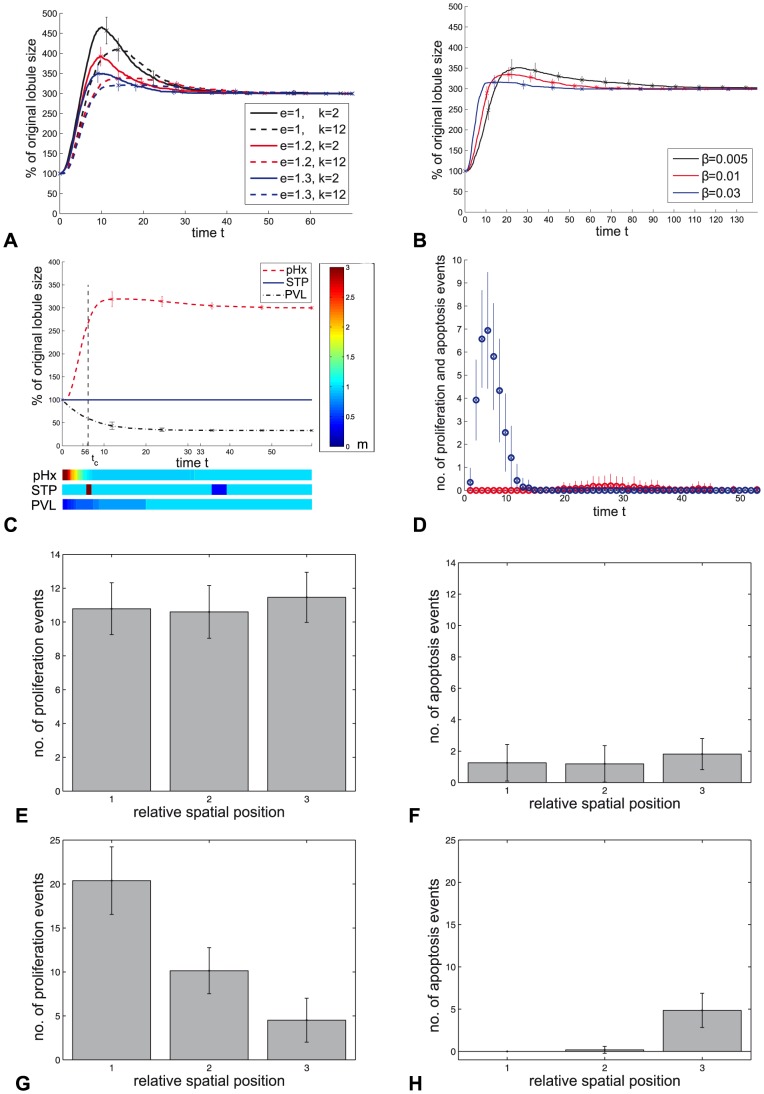
Simulation results of the ML model. **A** Temporary progression of the lobule size in dependence of the potential additional degradation 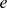 and the tolerance range for changes form the normal buffer level 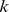. **B** Temporary progression of the lobule size in dependence of 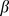, the sensitivity to deviations from the normal value 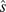. **C** Temporary progression of the lobule size in the simulation with parameters 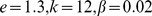 (blue dashed line in **A**) in the scenarios (pHx), (STP) and (PVL) as given in Table 1. The metabolic load in the signal layer is illustrated by a color-code for the different scenarios. The time point at which compensatory growth is 90% completed is marked with 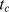. **D** The frequency distribution of proliferation (blue) and apoptosis (red) events for scenario (pHx) is depicted as number of events per time point. **E**-**H** Spatial distribution of proliferation and apoptosis events in the ML model simulation. For each time point, the space of the hepatocyte layer is equally divided into three sections displayed on the horizontal axis. The events are recorded for each section over the entire simulation time and are depicted as number of proliferation events for different relative spatial positions. **E** and **F** display the spatial distribution of proliferation (**E**) and apoptosis (**F**) events in the ML model with a uniform signal layer. **G** and **H** display the spatial distribution of proliferation (**G**) and apoptosis (**H**) events in the ML model with a non-uniform signal layer.

In Figure 6 the simulation results of the metabolic load (ML) model are summarized. First, results of the sensitivity analysis for the free parameters 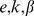 in the scenario (pHx) are reported. It shows that the potential additional degradation 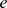, as well as the tolerance range for changes from the normal buffer level 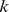, have an effect on the maximal HC number. The result for values of 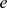 and 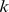 in the ranges 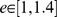 and 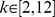 is depicted in the Figure 6A. With increasing 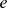 or 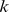 the maximum HC number during the simulation decreases. In any of the chosen cases, the final HC number equals three times the initial HC number. If 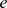 is chosen greater than 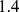 the final HC number remains below this level. The parameter 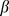, the sensitivity to deviations from the normal value 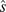, has an effect on the temporal progression as well as on the maximal HC number, see Figure 6B. The value of 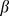 is varied in the range 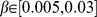. The analysis shows that the parameter set for which a result is observed that is in accordance with experimental observations is not unique. The parameter set which is chosen for the simulations of the scenarios is 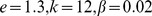.

In the simulations for (pHx), the HC number triples for the ML model with uniform signal layer as well as for the model with a non-uniform signal layer. The increase in the HC number is rapid and the final HC number is stable. Compensatory growth is completed to an extent of 90% within 6.2 time units, see Figure 6C. The metabolic load in the signal layer normalizes correspondingly, see Figure 6C horizontal bar below.

The growth curve is characterized by an overshooting in the HC number. The extent of the overshooting is 6.5% and it is corrected by apoptosis events in a later phase of the simulation.

The comparison between simulations with a uniform distribution of metabolites and simulations with a gradient distribution shows that the course of the growth curves is not altered by the existence of a gradient. Figure 6E-H shows the spatial distribution of proliferation and apoptosis events in the ML model simulation. This distribution correspond to the spatial distribution of the metabolites. The frequency of proliferation and apoptosis events in the simulation is depicted in Figure 6D. In the ML model with a uniform signal layer, the proliferation and apoptosis events are uniformly distributed along the sinusoid, see Figure 6E and 6F. In case of the ML model with a non-uniform signal layer, proliferation events are observed more frequently near the signal source, see Figure 6G. Apoptosis events occur only at greater distance from the signal source, see Figure 6H.

There is no change in HC number in case of (STP), see Figure 6C. The model is thus robust against (STP) as chosen here. Perturbations applied for a greater time span lead to proliferation and subsequent apoptosis (data not shown)

In the simulation of (PVL) a decrease in HC number is observed, that scales to the degree of metabolic load decline. During the simulation of (PVL) the HC number reaches a stable state and the metabolic load normalizes correspondingly, see Figure 6C.

#### Comparison between simulation results and experimental observations

The simulation results are compared to the criteria (O1)–(O5), which are derived from experimental observations. The results are summarized in the Table 2. It can be concluded that, the HD as well as the ML model provide a mechanism for size regulation. In the (pHx) scenario, the final HC number agrees with the experimental observations in both models. Furthermore, both models can be parametrized such that the time until the final HC number is achieved is within an experimentally plausible time span. Also in the (PVL) scenario the simulation results are in accordance with the experimental observations. However, only the ML model is robust against short-term perturbations. This can not be achieved in the HD model. A further criterion to distinguish is the observation that over-shooting and subsequent apoptosis at a later stage occur only in the in the ML model.

**Table 2:** Model evaluation on the basis of experimental data.

Evaluation data 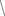 Model	HD	ML
(O1) Tripling of HC number in case of (pHx)	yes	yes
(O2) Time until 90% of the final HC number is achieved can be scaled to 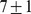 time units	yes	yes
(O3) Decrease in HC number at a later simulation stage	no	yes
(O4) Robust against short-term perturbations	no	yes
(O5) Decrease in HC number in case of (PVL)	yes	yes

The simulation results are evaluated on the basis of the criteria (O1)–(O5), which are based on experimental observations, see the Analysis.

## Discussion

The remarkable capacity of the liver to precisely restore its original size is studied here choosing partial hepatectomy as biological reference system. Liver size is modulated by hepatocyte proliferation and apoptosis. Two portal blood flow related hypotheses, the metabolic load and the hemodynamics hypothesis are discussed in the literature as trigger of proliferation and apoptosis. This is the first study that abstracts the principle feedback mechanisms for both hypotheses and evaluates them with the help of mathematical modeling.

The simulation results of the developed mathematical models, the metabolic load and the hemodynamics model, are compared to experimental data on the organ scale. For this purpose, a 70% partial hepatectomy and a 70% portal vein ligation were performed. Liver weight adaptation was calculated based on the relative fraction of the weight of the explanted liver respectively the relative liver-to-body weight ratio. Data regarding the weight of the liver require the explantation of the liver. In consequence, data for each observation time point was generated using different animals. This can be considered as a study limitation, nevertheless assessment of liver weight adaptation using the same animal would require a highly precise imaging modality enabling the repeated investigation under anesthesia. Devices for small animal imaging are principally available, but only in highly selected centers. Furthermore, repeated imaging requires repeated anesthesias which puts an animal recovering from major surgery at a substantial vital risk.

On the basis of the comparison to experimental data, it is concluded that the metabolic load model is preferred to the hemodynamics model. The metabolic load model provides a simple and plausible mechanism that leads to the characteristic growth curve progression seen in the experiments and in particular to robustness against fluctuations. As a consequence, the hemodynamics model alone is not sufficient to describe all characteristics of liver size regulation. Nevertheless, both mechanisms may contribute to the process of liver regeneration, although to differing degrees. In any case, the cellular capability to buffer is realized as a key mechanism for understanding liver size regulation.

The key difference between the two mechanisms is in the cellular response to the different proliferation triggering signals. In particular, the cellular capability to buffer discriminates both mechanisms. Concerning the hemodynamics model, the integration of a buffer mechanism as in the metabolic load model seemed not reasonable in the current state of knowledge and is therefore not considered in this study. It should be noted that there might be temporal delay between stimulus and cellular response due to the activation of signaling pathways. However, this temporal delay would apply for the hemodynamics, as well as for the metabolic load model in the same way and is therefore neglected.

It shows that the functional demand imposed on an organ could provide the basis for a size regulation mechanism. A mechanism based on the functional demand needs to solve the trade-off between a rapid response in case the organ size requires an adjustment and robustness in case of a short-term fluctuation of the demand. The present study presents a solution to this trade-off, which might be applicable to the size regulation in organ-extrinsically regulated organs in general.

The established model framework is a one-dimensional cell-based model extended by a layer representing the strength of the blood-borne signal. It is well-suited for the focus of this study which is to compare the potential effects of the principle hemodynamics and metabolic load feedback mechanism. At the same time the model provides the basis for future studies as it can be easily extended. In the literature, there are intracellular mechanisms proposed that could account for the triggering of hepatocyte proliferation by metabolites [4] or shear stress [25], respectively. Future work could consider different intracellular processes and their implications on the feedback mechanisms. These intracellular processes can be implemented by integration of a system of ordinary differential equations into each cell. Furthermore, the integration of more sophisticated blood flow models could be subject of future work. This could be done in order to test the effects of deviations from the modeling assumptions made here. Additionally, the consideration of arterial perfusion and thus the oxygen supply may constitute a first model extension. The focus in this study is on the portal blood supply. However, the contribution of arterial blood supply is particularly important as regards the oxygen supply of the liver. The oxygen supply seems to be crucial in case of an extended partial hepatectomy [41].

The findings of this study provide suggestions for future experimental work that can help to gain further clarity. The experimental data regarding an intermediate over-shooting in the regenerative response after partial hepatectomy is ambiguous. One of the problems might be that the over-shooting could be considerable small and the reliable detection of small differences in experimental studies would require large animal numbers. Nevertheless, as over-shooting is only observed in the metabolic load model, a detailed study on this phenomena would provide an argument for one of the models. In the metabolic load model, parameters that describe the sensitivity of hepatocyte to metabolic alterations, as well as the parameter that describes the ability of hepatocytes to adapt to these alterations influence the extent of over-shooting of the regenerative response. An according manipulation of equivalent characteristics of hepatocytes in experiments could help to enhance a potential over-shooting and therefore provide more clarity. Furthermore, model parameters that influence the temporal progression of lobule growth are identified in both models. In the hemodynamic model the according parameter is the sensitivity of hepatocytes to changes in the shear stress level. In the metabolic load model parameters that describe the sensitivity of hepatocyte to metabolic alterations, as well as the parameter that describes the ability of hepatocytes to adapt to these alterations influence the temporal progression. An according effect could be tested in experiments. The robustness against fluctuations is identified as an important criterion of the liver size regulation mechanism. In order to investigate experimentally the implications of the two hypotheses to the robustness of liver size, one should test the growth response on different hemodynamical or metabolic load stimuli, which vary in their duration and extent. Additionally, experiments that influence the portal blood flow like the portal vein ligation can be utilized to characterize the role of the metabolic load and shear stress hypotheses. If it is assumed that after portal vein ligation part of the metabolites circulate back to the liver via the hepatic artery [19] due to a systemic overload, the shear stress and the concentration of metabolites in the blood are not altered to the same extent by such a ligation. Therefore, a portal vein ligation of different extents or a combination of portal vein ligation and partial hepatectomy would help to rank the effect of both hypotheses for liver size regulation. With regards to the metabolic load model, spatially resolved data on the lobule scale for proliferation and apoptosis events could help to characterize the concentration gradient of the potential metabolites or even argue against the metabolic load model as proposed here. Knowing the characteristics of the concentration gradient in turn could help to specify metabolites that could play a major role. This specification is a precondition for a quantification of the metabolic load model. In order to quantify the hemodynamics model, measurements of hemodynamic factors on the sinusoidal scale in the biological model rat or mice are required.

Understanding the crucial factors for liver size regulation is of scientific and clinical importance. Precise prediction of the regenerative capacity and the time course would help to better plan for two stage liver resection procedures such as portal vein embolisation prior to extended resection and the associating liver partition and portal vein ligation for staged hepatectomy (ALLPS) procedure. Furthermore, assuming that one knows which metabolites are crucial for liver size regulation, one could use this knowledge to purposefully modulate donor liver size before transplantation or to accelerate the size recovery of the remaining liver after partial hepatectomy.

## Supporting Information

Figure S1 **One-dimensional mathematical model describes the growth of a liver lobule.** The liver, a cross-section of liver lobules and a row of hepatocytes along one sinusoid in a liver lobule are sketched. The individual hepatocytes are marked in gray and the sinusoid between portal vein (PV) and central vein (CV) is marked in blue. The latter row of hepatocytes serves as one-dimensional liver lobule representation. The number of hepatocytes in the one-dimensional liver lobule can be utilized to approximate the lobule size and thus the liver size.
(EPS)

Figure S2
**The growth rate per hepatocyte** 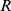 **depends on the shear stress signal strength** 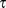 **.** Positive 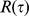 is referred to as proliferation rate and negative 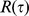 as apoptosis rate. The sensitivity to deviations from the normal value 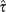 is controlled by slope 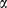. The growth rate per hepatocyte is limited by 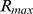 and 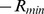.(EPS)

Figure S3
**Specifications for the ML model.** **A** Schematic representation of the model with a discretized non-uniform signal layer with a periodic boundary. Metabolites 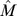 enter the signal layer at 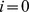 and are transported towards 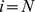. The external state at the position 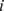 for a fixed time point 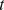 is denoted by 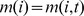. Metabolites are partly taken up by the hepatocytes in the hepatocyte layer. The internal state at the position 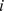 for a fixed time point 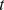 is denoted by 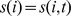. **B** The amount of metabolites a hepatocyte buffers 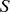 depends on the metabolic load 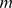, see (6) and (7). The amount hepatocytes buffer under normal metabolic load conditions is 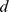. The specification of 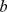 is described in the section Mathematical model. The spare buffer capacity is 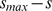. **C** The amount of metabolites a hepatocyte degrades 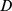 depends on the buffer level 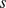, see (8). Under normal metabolic load conditions 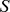 equals 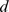. The tolerance range for changes form the normal buffer level 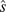 is 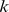 and the potential additional degradation is specified by 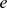. **D** The growth rate per hepatocyte 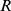 depends on the intracellular buffer level 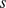, see (9). The sensitivity to deviations can be modulated by the slope 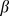 of the function. The growth rate per hepatocyte is limited by 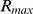 and 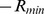.(EPS)

Dataset S1 **Experimental raw data.** Experimental raw data comprises data for liver regeneration after 70% partial hepatectomy in rats, experimental data for liver lobe size adjustment after portal vein ligation in rats and proliferation index for liver regeneration after 70% partial hepatectomy in rats.
(PDF)

Text S1 **Stability analysis for the steady states of approximating ordinary differential equation models.**
(PDF)
