# Supplementary material for: How Does a Single Cell Know When the Liver Has Reached Its Correct Size?
Source: PLoS One. 2014 Apr 1;9(4):e93207. doi: 10.1371/journal.pone.0093207 (PMC3972176; doi:10.1371/journal.pone.0093207)
Supplement: Dataset S1 — Experimental raw data. Experimental raw data comprises data for liver regeneration after 70% partial hepatectomy in rats, experimental data for liver lobe size adjustment after portal vein ligation in rats and proliferation index for liver regeneration after 70% partial hepatectomy in rats. (PDF) [file pone.0093207.s004.pdf]

Animal-Nr.	OP	OB-Time	BW(g)	remnant li	LL/BW(%)	AVE%	SD	fraction of liver	SD1
1	70%PH	24h	244	3.52	1.44				
2	70%PH	24h	292.5	5.29	1.81				
3	70%PH	24h	309	4.56	1.48				
4	70%PH	24h	319.5	4.92	1.54				
5	70%PH	24h	297.5	4.42	1.49				
6	70%PH	24h	319	4.02	1.26	**1.50**	**0.178**	0.455	0.054
7	70%PH	48h	265	5.78	2.18				
8	70%PH	48h	283	6.02	2.13				
9	70%PH	48h	297	5.95	2.00				
10	70%PH	48h	278	5.89	2.12				
11	70%PH	48h	282	5.65	2.00				
12	70%PH	48h	269	5.86	2.18	**2.10**	**0.081**	0.637	0.024
13	70%PH	72h	284	6.89	2.43				
14	70%PH	72h	263	7.35	2.79				
15	70%PH	72h	278	7.02	2.53				
16	70%PH	72h	280	7.25	2.59				
17	70%PH	72h	282.5	7.25	2.57				
18	70%PH	72h	286	7.21	2.52	**2.57**	**0.123**	0.779	0.037
19	70%PH	7d	273	8.05	2.95				
20	70%PH	7d	248	8.325	3.36				
21	70%PH	7d	250	7.865	3.15				
22	70%PH	7d	309	8.67	2.81				
23	70%PH	7d	299.5	7.77	2.59				
24	70%PH	7d	311	7.25	2.33	2.86	0.371	0.867	0.112
49	Sham-OP	24h	288	9.85	3.42				
50	Sham-OP	24h	291.5	8.72	2.99				
51	Sham-OP	24h	284.5	9.92	3.49				
52	Sham-OP	24h	300	7.716	2.57				
53	Sham-OP	24h	263.5	7.175	2.72				
54	Sham-OP	24h	279	7.87	2.82	3.00	0.376		
55	Sham-OP	48h	285	10.09	3.54				
56	Sham-OP	48h	270.5	9.1	3.36				
57	Sham-OP	48h	271.5	8.62	3.17				
58	Sham-OP	48h	295	9.39	3.18				
59	Sham-OP	48h	299	9.56	3.20				
60	Sham-OP	48h	299.5	10.3	3.44	3.32	0.155		
61	Sham-OP	72h	298.5	10.45	3.50				
62	Sham-OP	72h	296	10.38	3.51				
63	Sham-OP	72h	298.5	10.62	3.56				
64	Sham-OP	72h	274.5	10.045	3.66				
65	Sham-OP	72h	239.5	9.7	4.05				
66	Sham-OP	72h	288	8.765	3.04	3.55	0.323		
67	Sham-OP	7d	262	9.735	3.72				
68	Sham-OP	7d	255	11.247	4.41				
69	Sham-OP	7d	251.5	9.486	3.77				
70	Sham-OP	7d	331	8.447	2.55				
71	Sham-OP	7d	350.5	9.389	2.68				
72	Sham-OP	7d	335	9.646	2.88	3.33	0.741	3.30	

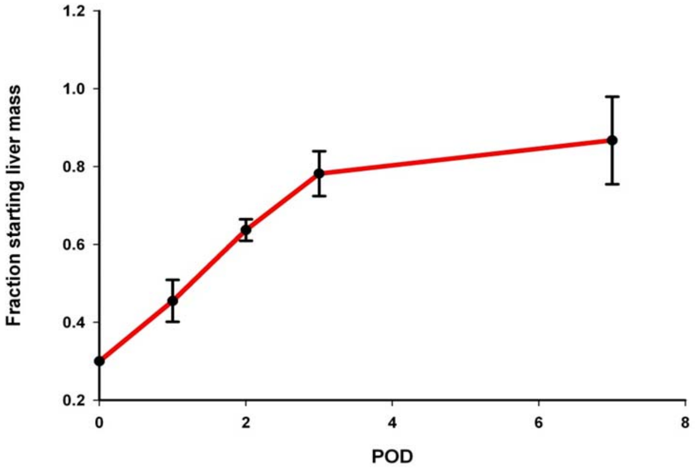

Graph produced by Sigmaplot 10.0

Animal ID	OP	servati	BW	RL	Remnant	LLL+ML	CL	LLL+ML%	AVE(1)	SD(1)	CL%	AVE(2)	SD(2)	RL%	AVE(3)	SD(3)
25	70%PVL	24h	253.5	1.680	7.500	4.61	0.76	1.819	1.900	0.087	0.300	0.304	0.009	0.663		
26	70%PVL	24h	269	2.170	8.860	5.45	0.85	2.026			0.316			0.807		
27	70%PVL	24h	282.5	1.800	8.350	5.23	0.84	1.851			0.297			0.637		
28	70%PVL	24h	263	1.851	8.780	5.03	0.79	1.913			0.300			0.704		
29	70%PVL	24h	268	1.848	8.763	4.87	0.79	1.817			0.295			0.690		
30	70%PVL	24h	264	1.870	9.012	5.22	0.83	1.977			0.314			0.708	0.701	0.058
31	70%PVL	48h	268	2.860	8.593	4.259	0.891	1.589	1.507	0.054	0.332	0.354	0.029	1.067		
32	70%PVL	48h	270	2.640	8.284	4.173	0.917	1.546			0.340			0.978		
33	70%PVL	48h	270	2.651	8.575	4.093	1.107	1.516			0.410			0.982		
34	70%PVL	48h	281	2.523	8.650	4.051	0.951	1.442			0.338			0.898		
35	70%PVL	48h	286	2.566	8.672	4.21	0.981	1.472			0.343			0.897		
36	70%PVL	48h	278	2.485	8.564	4.108	1.006	1.478			0.362			0.894	0.953	0.069
37	70%PVL	72h	305.5	3.683	10.372	3.89	1.509	1.273	1.299	0.143	0.494	0.468	0.040	1.206		
38	70%PVL	72h	299	2.796	8.970	3.857	1.463	1.290			0.489			0.935		
39	70%PVL	72h	290.5	3.292	10.356	4.589	1.351	1.580			0.465			1.133		
40	70%PVL	72h	274	3.611	9.603	3.445	1.416	1.257			0.517			1.318		
41	70%PVL	72h	250	2.824	7.664	3.023	1.071	1.209			0.428			1.130		
42	70%PVL	72h	256	3.260	8.007	3.035	1.057	1.186			0.413			1.273	1.166	0.136
43	70%PVL	7d	259	5.400	10.296	1.954	2.072	0.754	0.615	0.082	0.800	0.737	0.109	2.085		
44	70%PVL	7d	270.5	4.905	8.456	1.431	1.652	0.529			0.611			1.813		
45	70%PVL	7d	264.5	4.921	9.808	1.758	2.116	0.665			0.800			1.860		
46	70%PVL	7d	271	4.824	8.642	1.64	2.176	0.605			0.803			1.780		
47	70%PVL	7d	286	4.916	8.546	1.641	1.982	0.574			0.693			1.719		
48	70%PVL	7d	279	5.068	8.620	1.57	1.993	0.563			0.714			1.816	1.846	0.126
49	Sham-OP	24h	288	1.73	9.85	6.72	0.78	2.333	2.086	0.287	0.271	0.245	0.028	0.601		
50	Sham-OP	24h	291.5	1.74	8.72	6.35	0.75	2.178			0.257			0.597		
51	Sham-OP	24h	284.5	1.77	9.92	6.79	0.69	2.387			0.243			0.622		
52	Sham-OP	24h	300	1.68	7.716	4.793	0.778	1.598			0.259			0.560		
53	Sham-OP	24h	263.5	1.234	7.175	5.223	0.505	1.982			0.192			0.468		
54	Sham-OP	24h	279	1.571	7.87	5.68	0.69	2.036			0.247			0.563	0.569	0.055
55	Sham-OP	48h	285	1.71	10.09	7.11	0.71	2.495	2.182	0.208	0.249	0.236	0.013	0.600		
56	Sham-OP	48h	270.5	1.73	9.1	6.28	0.67	2.322			0.248			0.640		
57	Sham-OP	48h	271.5	1.8	8.62	5.92	0.63	2.180			0.232			0.663		
58	Sham-OP	48h	335	1.894	9.36	6.335	0.744	1.891			0.222			0.565		
59	Sham-OP	48h	339	1.748	10.12	7.064	0.824	2.084			0.243			0.516		
60	Sham-OP	48h	320.5	1.711	9.54	6.795	0.708	2.120			0.221			0.534	0.586	0.058
61	Sham-OP	72h	298.5	1.858	10.045	7.019	0.737	2.351	2.409	0.287	0.247	0.259	0.061	0.622		
62	Sham-OP	72h	296	1.658	9.7	6.807	0.725	2.300			0.245			0.560		
63	Sham-OP	72h	298.5	1.547	8.765	6.192	0.543	2.074			0.182			0.518		
64	Sham-OP	72h	274.5	2.05	10.309	7.207	0.656	2.626			0.239			0.747		
65	Sham-OP	72h	239.5	1.793	9.832	6.865	0.882	2.866			0.368			0.749		
66	Sham-OP	72h	288	1.874	9.443	6.45	0.779	2.240			0.270			0.651	0.641	0.095
67	Sham-OP	7d	262	1.761	9.735	6.902	0.763	2.634	2.339	0.546	0.291	0.248	0.029	0.672		
68	Sham-OP	7d	255	1.811	11.247	8.008	0.664	3.140			0.260			0.710		
69	Sham-OP	7d	251.5	1.778	9.486	6.649	0.672	2.644			0.267			0.707		
70	Sham-OP	7d	331	1.422	8.447	6.064	0.719	1.832			0.217			0.430		
71	Sham-OP	7d	350.5	1.578	9.389	6.383	0.791	1.821			0.226			0.450		
72	Sham-OP	7d	335	1.789	9.646	6.575	0.757	1.963			0.226			0.534	0.584	0.129

POD	ML-LLL		CL		RL	
	AVE	SD	AVE	SD	AVE	SD
1	0.91121	0.042	1.241	0.037	1.233682442	0.1023
2	0.69066	0.026	1.502	0.123	1.624991596	0.1185
3	0.53919	0.059	1.809	0.156	1.818258886	0.2114
7	0.26291	0.035	2.972	0.441	3.161214151	0.2163

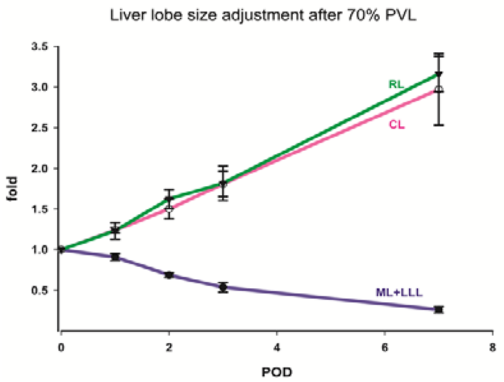

Graph produced by Sigmaplot 10.0

Group	ob-time(d)	PI of remnant liver lobes				AVE	AVE(PI)	SD
		RSL	RIL	SCL	ICL			
70%PH	1	0.33	0.34	0.24	0.25	0.290		
70%PH	1	0.35	0.36	0.30	0.28	0.323		
70%PH	1	0.21	0.20	0.22	0.20	0.208		
70%PH	1	0.22	0.21	0.21	0.2	0.210		
70%PH	1	0.28	0.25	0.25	0.21	0.248		
70%PH	1	0.33	0.34	0.26	0.27	0.300	0.263	0.049
70%PH	2	0.08	0.08	0.07	0.06	0.073		
70%PH	2	0.09	0.07	0.06	0.02	0.060		
70%PH	2	0.07	0.07	0.05	0.07	0.065		
70%PH	2	0.07	0.07	0.06	0.06	0.065		
70%PH	2	0.06	0.06	0.06	0.06	0.060		
70%PH	2	0.08	0.07	0.06	0.07	0.070	0.065	0.005
70%PH	3	0.07	0.06	0.05	0.05	0.058		
70%PH	3	0.04	0.04	0.03	0.04	0.038		
70%PH	3	0.03	0.04	0.03	0.02	0.030		
70%PH	3	0.06	0.05	0.08	0.07	0.065		
70%PH	3	0.04	0.04	0.03	0.04	0.038		
70%PH	3	0.02	0.02	0.01	0.02	0.018	0.041	0.018
70%PH	7	0	0	0	0	0.000		
70%PH	7	0	0	0	0	0.000		
70%PH	7	0	0	0	0	0.000		
70%PH	7	0	0	0	0	0.000		
70%PH	7	0	0	0	0	0.000		
70%PH	7	0	0	0	0	0.000	0.000	0.000

POD	PI		PI%	
	AVE1	SD1		
1	0.263	0.049	26.3	4.9
2	0.065	0.005	6.5	0.5
3	0.041	0.018	4.1	1.8
7	0.000	0.000	0	0

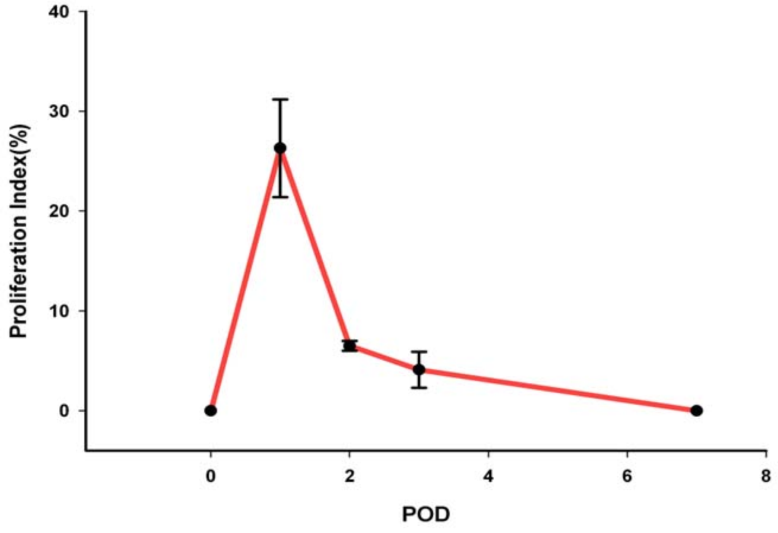

Graph produced by Sigmaplot 10.0
