# Supplementary material for: How Does a Single Cell Know When the Liver Has Reached Its Correct Size?
Source: PLoS One. 2014 Apr 1;9(4):e93207. doi: 10.1371/journal.pone.0093207 (PMC3972176; doi:10.1371/journal.pone.0093207)
Supplement: Text S1 — Stability analysis for the steady states of approximating ordinary differential equation models. (PDF) [file pone.0093207.s005.pdf]

Text S1. Stability analysis for steady states of approximating ordinary differential equation models.

Stability analysis for the ordinary differential equations system approximating the hemodynamics model

The only non-vanishing steady state of HD ordinary differential equations system is

$$N^* = c_P.$$

Put $u(N) := Nr\left(\frac{c_P}{N}\right)$ and observe that $\frac{dN(t)}{dt} = u(N(t))$ with

$$u(N) = \begin{cases} NR_{max}, & N \leq \frac{c_P}{R_{max}/a - \hat{\tau}}, \\ -NR_{min}, & N \geq \frac{c_P}{-R_{min}/\alpha - \hat{\tau}}, \\ (c_P - N\hat{\tau})\, a, & \text{otherwise.} \end{cases}$$

Evaluating the derivative of u at the steady state leads to

$$\left.\frac{du}{dN}\right|_{N=c_P} = -\hat{\tau}\alpha < 0,$$

which means that the steady state is stable.

Stability analysis for the ordinary differential equations system approximating the metabolic load model

The only non-vanishing steady state of the ML ordinary differential equations system is

$$(N^*, m^*, s^*) = (\hat{N}c_M, d, \hat{s}).$$

It is assumed that $s_{max} \gg 0$. Evaluating the Jacobian matrix at the steady state leads to

$$J = \begin{pmatrix} 0 & 0 & c_M\hat{N}\beta \\ \frac{-d}{\hat{N}c_M} & -1 & 0 \\ 0 & 1 & -1 \end{pmatrix}$$

With the help of the Routh Hurwitz criterion [1] statements about the stability can be made. Indeed, for the coefficients of the characteristic polynomial, it holds that

$$\begin{aligned} c_0 &= -\det(J) = \beta > 0 \\ c_1 &= \det(J_{11}) + \det(J_{22}) + \det(J_{33}) = 1 > 0 \\ c_2 &= -\mathrm{spur}(J) = 2 > 0. \end{aligned}$$

In addition,

$$c_1c_2 - c_0 = 2 - \beta > 0 \quad \text{for } \beta < 2.$$

Therefore, the steady state is stable for $\beta < 2$.

References

1. Gantmacher FR (2005) Applications Of The Theory Of Matrices. Dover Publications.
